# Late bleeding complications after mastectomy and the role of seroma aspiration: a retrospective 12-year cohort study

**DOI:** 10.1186/s12957-026-04340-4

**Published:** 2026-04-11

**Authors:** Anselm Tamminen, Riitta I. Aaltonen, Marko T. Ristola

**Affiliations:** 1 Department of Plastic and General Surgery, Turku University Hospital, Kiinamyllynkatu 4-8, Turku, 20521 Finland; 2 Department of Surgery, Faculty of Medicine, University of Turku, Turku, Finland

**Keywords:** Mastectomy, Seroma, Aspiration, Puncture, Antithrombotic medication, Hematoma, Bleeding complications

## Abstract

**Background:**

Surgical bleeding complications typically occur a few hours or days after the operation. Due to their rarity, late-onset bleeding complications are seldom discussed in the literature. Mastectomy is a common procedure, which is often associated with the need for repeated seroma aspirations. Although seroma aspirations have also been associated with complications, the area remains insufficiently studied. This study aimed to investigate the risk of late bleeding complications after mastectomy, especially those associated with seroma aspiration.

**Methods:**

Data from all patients undergoing simple mastectomy with or without axillary surgery for breast cancer between 2010 and 2022 at a single university hospital were collected. Medical records were reviewed for major bleeding complications and predisposing factors, especially the use of antithrombotic medications.

**Results:**

In total, 2,620 patients with 2,710 mastectomies were included in the study. Forty-five patients (1.7%) suffered a major bleeding complication. Twenty-four complications (53%) occurred after the first postoperative week; most (88%, 21/24) being preceded by a seroma aspiration. The median time from mastectomy to seroma aspiration-associated bleeding complication was 76 days (range 11–536 days). Upon arrival for treatment, 691 (26%) patients were taking antithrombotic medications. The risk for major bleeding complications related to seroma aspiration was 1.9% (13/691) and 0.4% (8/1929) in patients receiving and not receiving antithrombotic medications, respectively (*p* < 0.0001).

**Conclusion:**

A significant proportion of major post-mastectomy bleeding complications occur late and are associated with seroma aspiration, particularly in patients receiving antithrombotic therapy. The indication, technique, and setting of seroma aspiration in these high-risk patients should be carefully considered.

## Introduction

Breast cancer is the most common malignancy among women worldwide and its risk increases with age [1]. Older age is associated with conditions requiring antithrombotic medications, which predispose the patients to bleeding complications [2–4]. Additionally, elderly patients often undergo mastectomy, for example to avoid postoperative radiotherapy (RT) or the risk of reoperation after breast-conserving surgery (BCS), although mastectomy poses a higher risk for complications compared to BCS [5, 6].

One of the most common complications after breast cancer surgery is postoperative bleeding, which is usually reported to occur within hours or days after the procedure [7–9]. Surgical research usually reports complications occurring within the first 30 postoperative days, which is seemingly sufficient to cover bleeding complications [10]. However, patients undergoing mastectomy often develop subsequent seroma requiring aspirations [11]. The aspiration is performed through skin flaps containing blood vessels, which may be injured in the procedure. Although the risk of major complications may appear negligible, the mastectomy cavity is large, allowing for a large volume of bleeding. Especially in patients on antithrombotic medications, such complication may lead to significant bleeding that jeopardizes hemodynamic stability [12]. However, no prior study has systematically quantified the risk of major bleeding after seroma aspiration post mastectomy. A search performed in PubMed database (performed on February 12th, 2025, utilizing terms [“mastectomy” OR “breast surgery”] AND “seroma” AND [“puncture” OR “aspiration” or “drainage”] AND [“hematoma” OR “bleeding”]) produced only studies mentioning, but none investigating the subject [13].

To study this little-known topic and assess the risk for major bleeding complications related to seroma aspiration, we reviewed the data of all mastectomy patients treated at a single tertiary university hospital from 2010 to 2022. The primary outcome of this study was major postoperative bleeding after mastectomy. Early and late bleeding were analyzed as temporal stratifications of the primary endpoint, and bleeding events related to seroma aspiration were evaluated as a predefined subset analysis.

## Materials and methods

### Study population

Data for all patients who underwent simple mastectomy, defined as removal of the entire breast tissue, with or without axillary surgery, for breast cancer or ductal carcinoma in situ (DCIS) at a single tertiary hospital from 2010 to 2022 were collected from the Auria Clinical Informatics Register. Patients who underwent breast reconstruction at the time of surgery or those undergoing prophylactic mastectomy were not included.

### Surgical procedure

Mastectomy was mainly performed with energy-based instruments, based on the surgeon’s preference. Most surgeons preferred ultrasonic instruments, namely SonoSurg^®^ (Olympus Corporation, Tokyo, Japan), ThunderBeat^®^ (Olympus Corporation, Tokyo, Japan), and Harmonic Focus^®^ (Ethicon Inc., Cincinnati, OH, USA) and those surgeons not preferring ultrasonic instrument utilized predominantly an electrothermal bipolar technique based LigaSure Vessel Sealing System (Covidien Valleylab, Boulder, CO, USA).

Skin flaps were prepared to 5–10 mm in thickness. Axillary lymph node dissection (ALND) was performed if lymph node metastasis was biopsy-proven before the operation or the metastasis was detected in frozen section study of sentinel lymph node biopsy (SLNB). SLNB was performed using the triple technique (preoperative ^99m^Tc isotope injection, lymphoscintigraphy, and intraoperative blue dye injection). Frozen section analysis was performed on all sentinel lymph nodes until 2018, and only in selected cases thereafter, in accordance with the updated treatment guidelines.

The perioperative management of antithrombotic therapy varied by surgeon and by medication. Information on whether an antithrombotic agent affecting coagulation was discontinued or continued across surgery was recorded.

A single drain was placed, or two drains in bilateral mastectomies, each kept in place until daily exudate decreased to less than 80 ml, or for a maximum of seven days. For wound closure, the subdermal tissue was approximated using absorbable sutures, and the skin was closed with a continuous intracutaneous absorbable suture.

## Postoperative care

Patients had a postoperative follow-up appointment approximately three weeks after surgery. In cases of postoperative seroma formation, the patients were usually referred to primary health care for seroma aspirations. According to local practice, seroma aspiration is performed with the patient in the supine position, accessing the seroma cavity from its lateral aspect, typically using an 18-gauge, 40-mm needle. Ultrasound is not routinely used in case of clinically evident seroma. In case of major complications, the patients were referred to public specialized healthcare which in Finland is almost exclusively responsible for the management of such complications. The study was conducted at a tertiary university hospital managing all major surgical complications in the district, indicating that the complications that occurred are included in the data with few exceptions.

## Definitions

The primary outcome of the study was the occurrence of a major postoperative bleeding complication following mastectomy. A standardized definition for major bleeding complication was used [14]. Effectively, the criteria were fulfilled if the bleeding complication required reoperation or resulted in a decrease in hemoglobin level of at least 20 g/l.

In the absence of standardized criteria, bleeding complications were considered early if they were detected within the first postoperative week and late if detected thereafter. The temporal classification was applied to describe the timing of bleeding complications and to enable comparison between early and late complication events.

Because many seroma aspirations were performed in primary care and were incompletely documented, the number of individual aspiration episodes could not be reliably determined. Therefore, bleeding risk was estimated per mastectomy rather than per aspiration episode.

## Data collection

For each patient, age, body-mass index (BMI), ASA physical status classification (American Society of Anesthesiologists), and use of antithrombotic medication were recorded. The following data were collected in relation to mastectomy procedure: performing surgeon, performed axillary surgery, duration of the surgery, laterality of the operation and utilized surgical instrument. Information on all admissions to emergency department and to specialized health care related to breast cancer, performed surgical procedures, patient records with the complication diagnosis code (T81 in ICD-10), laboratory tests associated with bleeding complications (hemoglobin, hematocrit, and blood transfusions) was retrieved from the database and the accuracy of the data was verified from the electronic patient records. Patient records were reviewed throughout the entire study period to ensure that all events, including late events, were captured.

### Statistical analysis

The data were analysed using JMP 17 Pro (SAS Institute, Cary, North Carolina, USA) analysis software. For patients’ age and BMI, median and interquartile ranges were defined. Frequency tables for bleeding complications were generated. Univariable logistic regression analysis was used to identify predictors of postoperative bleeding complications, with early and late bleeding complications as the dependent variable in separate analyses. All patient and surgery-related variables described in the Data collection section were included in the analysis. The results are reported as odds ratios with 95% confidence intervals. A chi-square test was used for categorical variables, and a two-sample t-test or Wilcoxon rank-sum test was used for normally and non-normally distributed continuous variables, respectively.

The research protocol of the study was approved by the Hospital District of Southwest Finland (T537/2022). Data were analysed in a pseudonymized manner in accordance with institutional and national data protection regulations. This study is reported in accordance with the Strengthening the Reporting of Observational Studies in Epidemiology (STROBE) guidelines [15].

## Results

In total, 2,620 patients were included in the study. Bilateral mastectomy was performed in 93 patients, totalling 2,713 breasts at risk (Table 1). Operations were performed by 38 surgeons with varying experience on breast cancer surgery. Overall, 14 surgeons performed more than 50 mastectomies.

**Table 1:** Patient characteristics and information of antithrombotic medications. Values are n (%) unless otherwise indicated. (IQR= interquartile range, BMI = body-mass index, ASA = American Society of Anesthesiologists, DCIS = ductal carcinoma in situ, ALND = axillary lymph node dissection, SLNB = sentinel lymph node biopsy, BCS = breast conserving surgery *percentage of antithrombotic medications)

2620 patients	
Age (years), median (IQR)	67.9 (55.8–79.1)
BMI (kg/m^2^), median (IQR)	26.1 (22.9–30.1)
ASA Classification	
ASA Class 1	354 (14%)
ASA Class 2	1155 (44%)
ASA Class 3	1005 (38%)
ASA Class 4	98 (3.7%)
Information missing	8 (0.3%)
Antithrombotic medication	691 (26%)
-Acetylsalicylic acid	309 (45%)*
-Other antiplatelets	38 (5.5%)*
-Warfarin	166 (24%)*
-Direct oral anticoagulants	106 (15%)*
-Combination therapy (usually aspirin and dipyridamole)	20 (2.9%) *
-Low-molecular-weight heparin	29 (4.2%)*
Bilateral mastectomy	93 (3.4%)
Mastectomy performed as reoperation after BCS	279 (11%)
Patients with invasive breast cancer (in either breast)	2443 (93%)
Patients with DCIS (no invasive cancer in either breast)	177 (6.8%)
Axillary surgery	
ALND	1437 (54%)
SLNB	1047 (40%)
None	136 (5.1%)
Duration of surgery (min, IQR)	102 (80–120)

**Fig. 1:**
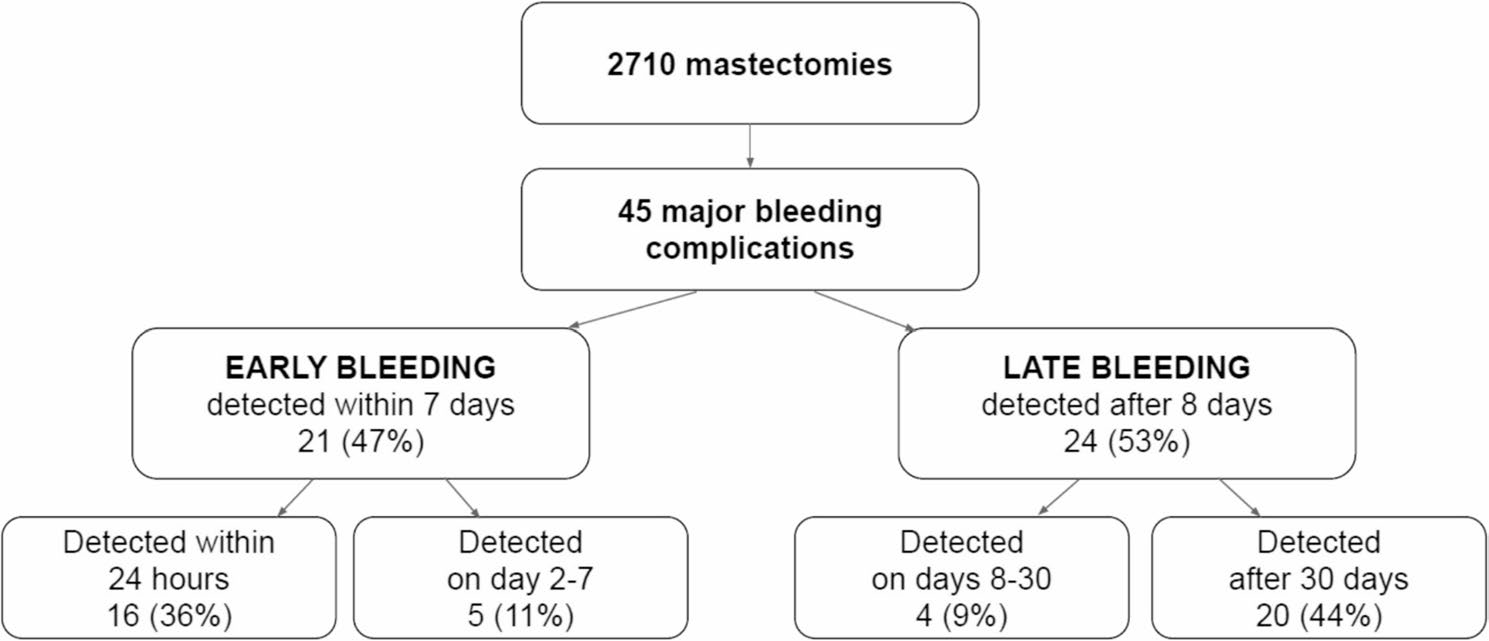
Temporal distribution of major bleeding complications after mastectomy

During the entire study period, 45 patients (1.7%, 95% CI 1.3%−2.3%) suffered a major bleeding complication (Fig. 1). Three patients were managed without surgical intervention and 42 underwent surgery. One patient presenting with a bleeding complication had no comparison value for hemoglobin and did not undergo reoperation, so the patient did not meet the criteria for major bleeding complication. As shown in the Fig. 1, less than half of the patients (47%, 21/45) suffered the complication during the first postoperative week, with the majority (76%, 16/21) of these occurring within 24 h of the initial operation.

In the statistical analysis, the risk factors for early (Table 2) and late bleeding complications (Table 3) differed.

**Table 2:** Comparison of patients suffering early postoperative bleeding complications to the patients not suffering the complication. Values are n (%) unless otherwise indicated. #Odds ratio could not be estimated due to zero events. (IQR= interquartile range, BMI = body-mass index, ASA = American Society of Anesthesiologists, ALND = axillary lymph node dissection, SLNB = sentinel lymph node, CI = confidence interval)

Patients with early bleeding complication	Yes (*n* = 21)	No (*n* = 2599)	*p*-value	Odds Ratio (95% CI)
Age (years), median (IQR)	67.2 (63.0–82.9.0.9)	67.9 (55.8–86.1)	*p* = 0.31	1.01 (0.98–1.04) per unit
BMI (kg/m^2^), median (IQR)	25.1 (22.9–27.1)	26.1 (22.9–30.1)	*p* = 0.21	0.96 (0.87–1.04) per unit
ASA Classification				
ASA Class 1	2 (9.5%)	352 (14%)	*p* = 0.81	reference
ASA Class 2	8 (38%)	1147 (21%)		1.23 (0.26–5.81)
ASA Class 3	10 (48%)	995 (38%)		1.77 (0.39–8.11)
ASA Class 4	1 (4.8%)	97 (3.7%)		1.81 (0.16–20.2)
Antithrombotic medication	9 (43%)	682 (26%)	*p* = 0.09	2.10 (0.88–5.02)
No antithrombotic medication	12 (57%)	1917 (74%)		reference
Antithrombotic medication discontinued perioperatively				
Yes	4 (1.3%)	283 (99%)	*p* = 0.93	1.10 (0.27–4.20)
No	5 (1.3%)	390 (99%)		reference
Not recorded	0	10		#
Axillary surgery				
ALND	12 (57%)	1425 (55%)	*p* = 0.59	0.94 (0.37–2.38)
SLNB	9 (43%)	108 (40%)		reference
None	0 (0%)	136 (5.2%)		#
Bilateral mastectomy	3 (14%)	90 (3.5%)	*p* = 0.02*	4.64 (1.34–16.1)
Unilateral mastectomy	18 (86%)	2509 (97%)		reference
Ultrasonic instrument	10 (48%)	2205 (85%)	*p* < 0.001*	0.16 (0.07–0.39)
Any other instrument	11 (52%)	394 (15%)		reference
Duration of surgery (min, IQR)	93 (78–129)	102 (80–120)	*p* = 0.96	1.00 (0.99–1.01) per unit

**Table 3:** Comparison of patients suffering late postoperative bleeding complications to the patients not suffering the complication. Values are n (%) unless otherwise indicated. #Odds ratio could not be estimated due to zero events. (IQR= interquartile range, BMI = body-mass index, ASA = American Society of Anesthesiologists, ALND = axillary lymph node dissection, SLNB = sentinel lymph node, CI = confidence interval)

Patients with late bleeding complication	Yes (*n* = 24)	No (*n* = 2596)	*p*-value	Odds Ratio (95% CI)
Age (years), median (IQR)	75.6 (64.6–82.4)	67.8 (55.8–79.1)	*p* = 0.007*	1.04 (1.01–1.07) per year
BMI (kg/m^2^), median (IQR)	26.8 (22.2–31.8)	26.1 (22.9–30.1)	*p* = 0.64	1.02 (0.95–1.09) per unit
ASA Classification				
ASA Class 1	3 (13%)	351 (14%)	*p* = 0.02*	reference
ASA Class 2	5 (21%)	1150 (44%)		0.48 (0.11–2.03)
ASA Class 3	13 (54%)	992 (38%)		1.41 (0.39–5.09)
ASA Class 4	3 (13%)	95 (13%)		3.56 (0.69–18.5)
Antithrombotic medication	13 (54%)	678 (26%)	*p* = 0.002*	3.34 (1.49–7.50)
No antithrombotic medication	11 (46%)	1918 (74%)		reference
Antithrombotic medication discontinued perioperatively				
Yes	9 (3.1%)	278 (97%)	*p* = 0.11	3.16 (1.02–11.8)
No	4 (1.0%)	391 (99%)		reference
Not recorded	0	10 (100%)		#
Axillary surgery				
ALND	16 (67%)	1421 (55%)	*p* = 0.77	1.31 (0.17–10.1)
SLNB	7 (29%)	1040 (40%)		0.94 (0.11–7.67)
None	1 (4.2%)	135 (5.2%)		reference
Bilateral mastectomy	2 (8.3%)	91 (3.5%)	*p* = 0.20	2.50 (0.58–10.8)
Unilateral mastectomy				reference
Ultrasonic instrument	21 (88%)	2194 (85%)	*p* = 0.68	1.28 (0.38–4.32)
Any other instrument	3 (13%)	402 (15%)		reference
Duration of surgery (min, IQR)	98 (85–113)	102 (79–120)	*p* = 0.64	1.00 (0.98–1.00.98.00)

Of the 24 bleeding complications that occurred after the first seven postoperative days, 21 (88%) were explicitly recorded as resulting from a preceding seroma aspiration. In two of the three patients in whom the bleeding complication could not be directly attributed to a preceding seroma aspiration, both had a history of prior seroma aspirations, but the hematoma was detected as an asymptomatic finding during routine follow-up. In these patients the hematoma was followed at first, but both ultimately required surgical treatment. The reoperations in these two patients were performed on postoperative days 158 and 2044, respectively.

The third patient suffered a postoperative surgical site infection. The patient’s wound was partially opened as part of the treatment for the infection, which caused major bleeding that necessitated surgical intervention, performed on the 13th postoperative day.

None of these three patients were receiving antithrombotic medications.

In the remaining 21 patients, the bleeding complication was explicitly attributable to a preceding seroma aspiration (Table 4). Typically, these complications occurred more often months rather than weeks after the operation (median 76 days, range 11–536 days), only three of them occurring within the first 30 postoperative days. Most of these patients (62%, 13/21) were receiving antithrombotic medications at the time of the bleeding complication, and the risk for bleeding complication substantially higher in these patients (13/691, 1.9%; 95% CI 1.1–3.2%) than in patients without such medications (8/1929, 0.4%; 95% CI 0.2–0.8%) corresponding to an absolute risk difference of 1.5% points (*p* < 0.0001).

**Table 4:** Characteristics of patients suffering from seroma aspiration-related bleeding complication. (ALND = axillary lymph node dissection, SLNB = sentinel lymph node biopsy)

Time from primary operation (days)	Patients age (years)	Body-Mass Index (kg/m^2^)	Bilateral Mastectomy	Axillary operation	Antithrombotic medication	Urgency classification of complication surgery	Hemoglobin level (g/l) at the time of diagnosis
11	57	21.2	No	ALND	no	Emergency	77
13	91	25.0	Yes	ALND (Bilateral)	enoxaparin	Emergency	73
20	70	28.6	No	SLNB	warfarin	Urgent	86
30	78	20.2	No	ALND	no	Urgent	82
49	64	24.2	No	ALND	acetylsalicylic acid	Urgent	-
52	91	28.0	No	ALND	dabigatran	Urgent	93
54	56	21.6	No	ALND	no	Urgent	-
55	82	34.9	No	ALND	combination of acetylsalicylic acid and clopidogrel	Emergency	85
57	69	42.8	Yes	ALND (Bilateral)	rivaroxaban	Urgent	113
69	81	28.5	No	ALND	acetylsalicylic acid	Urgent	-
76	89	32.4	No	ALND	apixaban	No surgery	90
89	81	30.9	No	ALND	no	Urgent	96
110	69	20.1	No	None	no	Elective	-
133	82	23.7	No	SLNB	acetylsalicylic acid	Elective	81
209	87	28.4	No	SLNB	acetylsalicylic acid	Elective	128
246	77	35.7	No	ALND	no	Elective	121
359	74	32.2	No	ALND	no	Elective	107
370	62	27.6	No	SLNB	no	Elective	-
425	66	21.7	No	ALND	acetylsalicylic acid	Elective	119
461	75	26.0	No	SLNB	acetylsalicylic acid	Elective	126
536	84	34.7	No	ALND	rivaroxaban	Elective	127

In detailed analysis, the complications related to seroma aspiration could be divided further into two distinct entities: In the twelve patients, whose bleeding complication was diagnosed within three months of the primary operation, the complication typically occurred shortly after the seroma aspiration, developed rapidly and required urgent reoperation. The volume of blood evacuated in reoperation was high (mean 480 ml, range 200–800 ml) and caused a significant drop in patients’ hemoglobin. Most patients did not have a comparison value for hemoglobin taken between the mastectomy and the bleeding complication, but the mean level hemoglobin measured within reoperation was 88 g/l (range 73–113 g/l), significantly lower than what would be expected in patients without complications.

In nine patients whose bleeding complication developed more than three months after surgery, conservative treatment was typically attempted initially, with a follow-up period of several days or weeks, before the decision to proceed to reoperation was made. The volume of hematoma evacuated in these patients was lesser (mean 244 ml, range 150–500 ml) and the hemoglobin levels were higher than in patients suffering an early bleeding complication. Actually, the hemoglobin levels in all but two patients undergoing reoperation remained within normal range, likely reflecting the slow progression of the hematoma and the body’s ability to compensate for gradual blood loss.

## Discussion

To date, seroma aspiration-associated bleeding complications after breast surgery have received little attention. To our knowledge, this is the first published scientific study to provide a quantitative estimate of the incidence of such complications. The present study reveals that these complications constitute a relatively high proportion of all bleeding complications following mastectomy. Actually, the number of bleeding complications related to seroma aspiration was higher than the number of bleeding complications detected within 24 h after the surgery, which is the most typical time frame of surgical bleeding complications [10]. The risk for seroma aspiration-related bleeding complications was shown to be elevated especially in patients receiving antithrombotic medications compared to patients without such medications.

### Timing of bleeding complications

Based on the results of this study, it can be observed that the timing of bleeding complications is clearly associated with their clinical presentation.

Approximately one third of bleeding complications occurred during the first postoperative day, which is consistent with previous literature [10]. Such bleeding events typically develop rapidly, and patients usually require urgent reoperation to control the bleeding.

Bleeding complications diagnosed within the first postoperative week rarely have any precipitating factor other than the surgery itself. Although this aspect was not specifically investigated in the present study, it may be speculated that this finding could be related to less pronounced or more slowly developing postoperative bleeding. In the case of mastectomy, smaller amounts of bleeding may go undiagnosed initially, which may be explained by the relatively large surgical cavity, such that only a substantial volume of bleeding becomes clinically apparent [12].

Bleeding complications occurring without an evident predisposing factor are rare after the first postoperative week. The most common cause of such bleeding is seroma aspiration. Based on the interpretation of the patient cohort in this study, it appears that bleeding episodes occurring after seroma aspiration within the first three months following surgery tend to be more severe than those occurring thereafter. This may be explained by the large size and continuity of the aspirated seroma cavity, which gradually decreases in size over time, and allows a larger volume of bleeding the earlier it develops after surgery.

Overall, it is noteworthy that bleeding complications associated with seroma aspiration developed considerably later than expected, with a median onset of 76 days after surgery. Surgical complications are conventionally recorded only within a limited postoperative follow-up period, for example 30 days, and as a result these bleeding complications fall outside the scope of outcomes captured in standard studies [10].

Currently, there are no well-established criteria in the existing literature for classifying postoperative bleeding complications as either early or late. The cut-off values used for timing of bleeding complications in the literature are variable and partly arbitrary [16]. The results of the present study may provide guidance in the context of mastectomy.

### Risk factors for bleeding complications

The risk factors for early bleeding complications were bilateral surgery and used instrument - with ultrasonic instruments having the lowest risk for bleeding. It has previously been shown that the use of ultrasonic instruments is associated with a lower risk of bleeding compared with other surgical instruments [9].

The risk of late bleeding complications was higher in patients receiving antithrombotic medication than in those not receiving such medication. Higher ASA class and advanced age were also statistically associated with bleeding complications and were strongly correlated with the use of antithrombotic medication. Given the limited number of outcome events (*n* = 45), multivariable modeling was not performed, and the reported associations should therefore be considered exploratory and unadjusted for potential confounding. Consequently, causality cannot be inferred. Although antithrombotic medication may represent a clinically plausible contributing factor, age and ASA class likely reflect underlying comorbidity and treatment indication. These findings should therefore be interpreted with caution and considered hypothesis-generating. This interpretation would also be consistent with previous literature [17].

### Clinical relevance of bleeding complications

Even infrequent bleeding events may have a significant impact on patient outcomes and healthcare utilization. They may necessitate reoperation, prolong hospital stay, increase the risk of infectious complications, and delay adjuvant oncologic treatments [18, 19]. As shown in the present study, early bleeding complications are associated with a significant decrease in hemoglobin level. Anemia and major surgical bleeding are known to predispose the patient to severe thromboembolic complications, such as stroke and myocardial infarction [20, 21]. A previous study has reported that 6% of patients with atrial fibrillation who experienced stroke while receiving antithrombotic medication had undergone surgical operation within the preceding 30 days [22]. The use of antithrombotic medications is associated with an increased risk of bleeding complications, as well as underlying conditions that elevate the risk of thromboembolic and ischemic complications [23, 24]. For these reasons, appropriate perioperative management of antithrombotic medication is of paramount importance.

### Practical implications

This study demonstrated that postoperative seroma, and particularly seroma aspiration, is associated with an increased risk of bleeding complications requiring surgical intervention. The treatment and prevention of seroma is a long-term issue, and there is no clear solution that stands out as the best [25, 26]. This study adds another perspective to the discussion, emphasizing the need to find either an effective preventive measure or treatment for seroma formation. When considering seroma aspiration, it should be evaluated what volume of seroma justifies aspiration in terms of the risk–benefit balance, and whether reducing the needle size or using ultrasound guidance could decrease the risk of complications. In addition, performing the aspiration in specialized care by an experienced professional should be considered especially in high-risk patients, such as elderly individuals and those receiving antithrombotic medication.

We consider that the magnitude of the post-mastectomy bleeding risk warrants acknowledging when considering the surgical treatment in patients taking antithrombotic medications. It is recognized that mastectomy is associated with a substantially higher risk of postoperative seroma formation compared with BCS [27]. In our view, this study provides additional support for favoring BCS also in older patients with comorbidities.

Notably, the risk for early bleeding complications was not elevated in the patients who were taking antithrombotic medications. This results from the use of ultrasound instruments in mastectomy, as it has been previously demonstrated that these instruments reduce the risk of bleeding complications, regardless of the use of antithrombotic medications [9, 28]. This is particularly relevant, as interruption of antithrombotic medication is associated with an increased risk of surgery-related thromboembolic complications [29]. This study provides additional evidence suggesting that, when energy-based instruments are used, interruption of antithrombotic medication may not be necessary in mastectomy.

### Limitations

This study has several limitations. First, due to the retrospective nature of the study, there may be missing information, and the accuracy of the data cannot be verified on all aspects, and a certain portion of bleeding complications may be missing from the data. However, it could be argued that this study therefore presents the minimum risk of bleeding complications. Almost all patients in this study underwent mastectomy with energy instruments, and therefore the data does not represent a typical group of mastectomy patients. Based on this study, the absolute risk of major bleeding complication following a single seroma aspiration cannot be directly estimated, since most aspirations were performed in primary healthcare, where records of all aspirations could not be retrieved. Consequently, bleeding risk could only be estimated per mastectomy and not per individual aspiration episode. To be able to evaluate the true risk of bleeding complication in seroma aspiration, a prospective follow-up study should be executed.

## Conclusions

A significant proportion of post-mastectomy bleeding complications present later than 30 days after the surgery and are usually associated with preceding seroma aspiration. The risk is elevated especially in patients taking antithrombotic medications. The indication, technique, and setting of seroma aspiration in these high-risk patients should be carefully considered.

## Data Availability

The data that support the findings of this study are available on request from the corresponding author. The data are not publicly available due to privacy or ethical restrictions.
